# Prediction of High-Risk Types of Human Papillomaviruses Using Statistical Model of Protein “Sequence Space”

**DOI:** 10.1155/2015/756345

**Published:** 2015-04-20

**Authors:** Cong Wang, Yabing Hai, Xiaoqing Liu, Nanfang Liu, Yuhua Yao, Pingan He, Qi Dai

**Affiliations:** ^1^College of Life Sciences, Zhejiang Sci-Tech University, Hangzhou 310018, China; ^2^College of Sciences, Hangzhou Dianzi University, Hangzhou 310018, China; ^3^Department of Gynecological Oncology, Zhejiang Cancer Hospital, Hangzhou 310022, China; ^4^College of Sciences, Zhejiang Sci-Tech University, Hangzhou 310018, China; ^5^Center for Systems Biology, University of Texas at Dallas, Richardson, TX 75080, USA

## Abstract

Discrimination of high-risk types of human papillomaviruses plays an important role in the diagnosis and remedy of cervical cancer. Recently, several computational methods have been proposed based on protein sequence-based and structure-based information, but the information of their related proteins has not been used until now. In this paper, we proposed using protein “sequence space” to explore this information and used it to predict high-risk types of HPVs. The proposed method was tested on 68 samples with known HPV types and 4 samples without HPV types and further compared with the available approaches. The results show that the proposed method achieved the best performance among all the evaluated methods with accuracy 95.59% and *F1*-score 90.91%, which indicates that protein “sequence space” could potentially be used to improve prediction of high-risk types of HPVs.

## 1. Introduction

Cervical cancer is one of the leading causes of cancer morbidity and mortality in women worldwide [1]. Approximately, 500,000 new cases of cervical cancer were diagnosed each year, with 280,000 deaths [2]. It has become the second most common cancer among women especially for developing countries [3, 4]. Some studies have shown that human papillomavirus (HPV) is strongly associated with cervical cancer, and some types of HPVs can cause abnormal tissue growth in the form of warts (papillomas) and some HPVs are associated with certain cancers and precancerous conditions [5–7].

Human papillomaviruses are icosahedral, nonenveloped particles that contain a small, double-stranded circular DNA of approximately 8000 nucleotide base pairs [8] and belong to the Papillomavirus family (papilloma, polyoma, and simian vacuolating viruses) [9]. The diameter of circular DNA is approximately 55 nm [10–13]. Up to now, there are more than 150 HPV types, and some new types will be identified when they have significant homology differences with the defined HPV types [14–16]. Epidemiologic studies have shown that genital human papillomaviruses have a strong relationship with cervical cancer, independent of other risk factors. According to their relative malignancy, the genital tract HPVs can be grouped into two or three types: low-risk type, intermediate-risk type, and high-risk types [17]. But HPVs are usually divided into two types in clinical association study: high-risk or low-risk types. Low-risk viral types are more closely related with low-grade lesions, and high-risk viral types are associated with high-grade cervical lesions and cancers [17]. High-risk type is composed of 20 HPV types, such as HPV-16, HPV-18, HPV-26, HPV-31, HPV-33, HPV-35, HPV-39, HPV-45, HPV-51–53, HPV-56, HPV-58, HPV-59, HPV-66, HPV-68, HPV-70, HPV-73, HPV-82, and HPV-85 [18]. HPV-16 and HPV-18 are responsible for about 62.6% and 15.7% of cervical cancers [19]. Therefore, discrimination of high-risk types of HPVs becomes one of the most important things for diagnosis and therapy of cervical cancers.

Because of the importance of the HPV types, many epidemiological and experimental methods have been proposed to identify them [5, 20–22]. They are mostly based on the polymerase chain reaction (PCR), a sensitive technique for the detection of very small amounts of HPV nucleic acids in clinical specimens. With rapid increasing of the HPV data in protein and DNA databank, there is a great need to develop some reliable and effective computational methods to predict the high-risk types of HPVs directly from the available data.

Recently, some research works found the correlations between these data and high-risk types of HPVs and proposed some computational methods to predict the high-risk types of HPVs. Eom et al. learned the most informative subsequence segment sets of DNA sequences and used genetic algorithm to classify the risk types of each HPV [23]. Joung et al. classified the risk type of HPVs based on the hidden Markov model and the support vector machines using the protein sequences [24, 25]. Park et al. proposed a classification of the risk type of human papillomavirus by decision tree [26]. Kim and Zhang introduced the string kernel and Gap-spectrum kernel to compute the distances of amino acids pairs and further used them to classify HPV risk types based on E6 protein sequences [7, 9]. Kim et al. proposed an Ensemble support vector machine to classify HPV risk types based on the differential subsequences of protein secondary structures [13]. Esmaeili et al. calculated Chou's pseudo amino acid composition of E6 protein sequences and used ROC to predict HPV risk types [27]. Alemi et al. analyzed the physiochemical properties of all early and late proteins in high- and low-risk HPV types and introduced support vector machines to classify high-risk HPV types based on receiver operating characteristic analysis [28].

These methods have achieved promising results in high-risk types of HPVs prediction, but challenges for information extraction of HPVs still remain. The widely used information of HPVs in high-risk type prediction is sequence-based or structure-based information from the given DNA or protein sequence, and the information from related proteins or family has not been explored until now. With this problem in mind, we presented a novel scheme to predict high-risk types of HPVs using word statistical model of protein sequence space and support vector machine. We first constructed a “sequence space” of the given protein sequence with help of mutation matrices. We then extracted the information of HPV from the protein “sequence space” with the proposed word statistical model. At last, the extracted information was fed into support vector machine to predict high-risk types of HPVs. Through several experiments, we want to address how well the proposed prediction method performed when comparing with the available ones and whether the prediction abilities of the proposed prediction method depends on the choice of the mutation matrices.

## 2. Materials and Methods

### 2.1. Datasets

All types of HPV share a common genomic structure which is arranged into the upstream regulatory region (URR) and eight open reading frames (ORFs) encoding the viral early and late genes [11]. URR contains long control region, TATA signal 1 and TATA signal 2. There are polyA signal 1 and polyA signal 2 between early and late genes. Late gene expression produces the structural proteins L1 and L2 [12], which assemble into the viral capsid structure, whereas early gene activity translates into the regulatory proteins E1, E2, E4, E5, E6, and E7. In this paper, we constructed seven datasets of HPV protein sequences: E1, E2, E4, E6, E7, L1, and L2, respectively. Here, we did not use HPV E5 because the lengths of its protein sequences are too small. All the HPV datasets were downloaded from the Human Papillomaviruses Compendium published by Los Alamos National Laboratory (LANL).

There are total 72 types of HPVs in each dataset, but some HPV sequences are missing in LANL. So we downloaded the missing sequences from taxonomy browser in the National Center of Biotechnology Information. For example, HPV 43, 67, 75, 76, 77, and 80 protein sequences are missing in L2 dataset; we obtained these sequences from taxonomy browser. But we could not find the missed sequences of the E4 dataset in the National Center of Biotechnology Information, so the total number of HPV sequences is 71 in the E4 dataset. Among HPV sequences, four sequences (HPV 26, 54, 57, and 70) are selected as the predicting data and others are the training data [13]. Here, HPV risk types are manually determined based on the HPV compendium, in which seventeen HPV types are classified as high-risk types (HPV 16, 18, 31, 33, 35, 39, 45, 51, 52, 56, 58, 59, 61, 66, 67, 68, and 72) and the remaining are low-risk types.

### 2.2. Construction of Protein “Sequence Space”

It is well known that there are over 20 amino acids and each one is different from the others. Mutation matrices represent the similarities among amino acids. Let *AA*_*i*_ and *AA*_*j*_ denote two amino acids from the set *Ω*, and their score was defined as follows:

(1) $$\begin{array}{c} S\left(AA_{i},AA_{j}\right)=\text{Mutation}\left(AA_{i},AA_{j}\right), \end{array}$$

where Mutation(*AA*_*i*_, *AA*_*j*_) represents the “normalized probability” that the amino acid *AA*_*i*_ mutates into the amino acid *AA*_*j*_. In evolutionary biology, the score describes the rate at which one amino acid in a protein sequence changes to other amino acids states over time. That is to say the sequence similarity depends on the amino acids' scores represented in above definition. Usually, two amino acids *AA*_*i*_ and *AA*_*j*_ are considered similar if their score is more than zeros. It is worth noting that the similarity between *AA*_*i*_ and *AA*_*j*_ is symmetric, but it is not a transitive relation. For example, *AA*_*i*_ is similar to *AA*_*j*_ and *AA*_*j*_ is similar to *AA*_*k*_, but *AA*_*i*_ is not similar to *AA*_*k*_.

Taking amino acids' scores into mind, we classified 20 amino acids into several overlapping classes. Here, star sets were introduced, in which the properties are known between vertices and center. Given an amino acid *AA*_*i*_, its star set was defined as follows:

(2) $$\begin{array}{c} \text{Star}S\left(AA_{i}\right)={⋃}_{\alpha \in \Omega }\text{sig}\left(S\left(AA_{i},AA_{\alpha }\right)\right)\cdot \alpha , \end{array}$$

where sig is a function that returns the sign of a number, indicating whether the number is positive or zero. If number is greater than zero, 1, otherwise, to zero. For example, 20 amino acids can be partitioned into several star sets based on PAM250 mutation matrix, which was presented in Table 1.

We wanted to go further with the star sets and found some related protein sequences that have a high similarity among them. Suppose *S*_1_ = *s*_1_^1^ *s*_2_^1^ ⋯ *s*_*n*_^1^ and *S*_2_ = *s*_1_^2^ *s*_2_^2^ ⋯ *s*_*n*_^2^ are two given protein sequences; they are related if they satisfy the following condition:

(3) $$\begin{array}{c} \forall s_{k}^{1}\in \text{Star}S\left(s_{k}^{2}\right),\;\forall s_{k}^{2}\in \text{Star}S\left(s_{k}^{1}\right),\;1\leq k\leq n. \end{array}$$

From the above definition, it is easy to note that if two protein sequences have more similar sequences, they should be more closely related. With help of definition of related sequences, we constructed the “sequence space” of given sequence *S* = *s*_1_ *s*_2_ ⋯ *s*_*n*_, denoted by SP_*S*_, as follows.

**Step 1 .**

Given a null-set, denoted by *ϕ*, add its star sets to *ϕ* and obtain protein “sequence space” SP_*S*_.

**Step 2 .**

A prefix *s*_1_ was added to *ϕ* and obtained its star set Star*S*(*s*_1_). We checked whether the star set of the prefix *s*_1_ is empty or not. If its star set is a nonempty set, we added a symbol “−” after Star*S*(*s*_1_) and updated the protein “sequence space” SP_*S*_.

**Step 3 .**

We repeated Step 2 until the end of the given sequence *S* = *s*_1_ *s*_2_ ⋯ *s*_*n*_ and obtained its protein “sequence space” SP_*S*_ as follows:

(4) $$\begin{array}{c} \text{S}{\text{P}}_{S}=\overset{n}{\underset{k=1}{⋃}}\left[{⋃}_{\alpha \in \Omega }\text{sig}\left(S\left(AA_{k},AA_{\alpha }\right)\right)\cdot \alpha \right]-. \end{array}$$

In the construction of the protein sequence space, all the protein sequences were closely related to the given protein sequence. That is to say all the information on the related proteins or family could be explored through the construction of the protein “sequence space.”

### 2.3. Word Statistical Model in Protein “Sequence Space”

Word statistical model is one of the most widely used methods for sequence analysis [29–32]. In this model, each sequence is first mapped into an *m*-dimensional vector according to its word frequencies, and sequence similarity can be measured by distance measures, such as Euclidean distance [33], Mahalanobis distance [34], Kullback-Leibler discrepancy [35], and Cosine distance [36]. When the words occurring in biological sequence are estimative probabilities rather than the frequencies, they are more readily optimized by more complex models, such as Markov model [37–39], mixed model [40], and Bernoulli model [41]. These complex models could be considered to be the modification of traditional word-based models.

A biological sequence can be described as a succession of symbols, and a word is a series of *k* consecutive letters in the sequence. For a sequence *S* = *s*_1_ *s*_2_ ⋯ *s*_*n*_, the count of its word *W*_*k*_ = *w*_1_ *w*_2_ ⋯ *w*_*k*_, denoted by *c*(*W*_*k*_), is the number of occurrence of the word *W*_*k*_ in the sequence *S*. Here, we constructed a word statistical model in protein “sequence space.” First of all, a position function of an occurrence of the word *W*_*k*_ was defined as follows:

(5) $$\begin{array}{c} {ℵ}_{i}\left(s_{i},w\right)=\left\{\begin{array}{cc} 1, & \text{if}\;\;s_{i}=w,\\ 0, & \text{otherwise}. \end{array}\right. \end{array}$$

The count of the word *W*_*k*_ in the protein “sequence space” can be defined from the random indicators of occurrence as follows

(6) ΦWk=∑i=1n−k+1∑α1∈StarSsi∑α2∈StarS(si+1)⋯∑αk∈StarSsi+kℵiα1,w1×ℵi+1α2,w2×⋯hhhhhh×ℵi+kαk,wk.

In order to eliminate the effects of space size, we normalized the word contents with the size of the space and got word frequencies of protein “sequence space,” denoted as *F*_*k*_^SP_*S*_^. Consider

(7) FkSPS=fSPSWk,1,fSPSWk,2,…,fSPSWk,Y=ΦWk,1∏i=1n−k+1∏j=1kStarSsi+j,hlΦWk,2∏i=1n−k+1∏j=1kStarSsi+j,…,hlΦWk,Y∏i=1n−k+1∏j=1kStarSsi+j,

where |Star*S*| is the size of the star set and *Y* is the total number of the words that appear in the protein “sequence space” SP_*S*_.

### 2.4. Prediction Algorithm

There are two types of HPV protein sequences: high-risk type and low-risk type. Let *Y* = [*y*_1_, *y*_2_,…, *y*_*n*_]^*T*^ denote the type labels of *n* samples, where *y*_*i*_ = *k* indicates the *i*th sample being risk type *k*, where *k* = 1,2 denotes two different risk types (*y*_*i*_ = 1 indicates the *i*th sample being high-risk type, and *y*_*i*_ = 2 indicates the *i*th sample being low-risk type). Let *x*_*ij*_ be the *j*th word frequency in protein “sequence space” for the *i*th sample, where *j* = 1,2,…, *m*;  *X* = (*x*_*ij*_)_*n*,*m*_ denotes all the statistical information of “sequence space” for all samples,

(8) X=index1index2⋯indexmx1x2⋮xnx11inx12in⋯inx1mx21inx22in⋯inx2n⋮in⋮in⋱in⋮xn1inxn2in⋯inxnm,

where *x*_1_, *x*_2_,…, *x*_*n*_ are *n* samples and *x*_*i*_ = [*x*_*i*1_, *x*_*i*2_,…, *x*_*in*_] and *x*_*i*_ ∈ *R* ^*m*^. With help of the support vector machine (SVM), the prediction problem of HPV types was formulated as follows:

(9) min⁡w,b,ξ     Jw,b,ξ=12wTw+C∑i=1nξisubject  to    yiwTφxi+b≥1−ξi, i=1,2,…,n,subject  to    ξi≥0, i=1,2,…,n,

where *w* is defined as a Linear combination of the set of nonlinear data transformations

(10) $$\begin{array}{c} w=\overset{n}{\underset{i=1}{\sum }}\alpha_{i}y_{i}\varphi \left(x_{i}\right), \end{array}$$

*b* is a bias term, *C* is a regularization metaparameter, and *ξ*_*i*_ denotes the training error for the *i*th sample. This optimization problem derived in a dual space can be written as

(11) max⁡α     Jα=max⁡α∑i=1nαimax⁡α     Jα=−12∑i=1  n∑j=1nαiαjyiyjφxiTφxjsubject  to    ∑i−1nαiyi=0, i=1,2,…,n,subject  to    0≤αi≤C, i=1,2,…,n.

In this paper, we used the Gaussian radius basis function kernel to calculate the *φ*(*x*_*i*_)^*T*^ *φ*(*x*_*j*_) instead of calculating either *φ*(*x*_*i*_) or *φ*(*x*_*j*_) explicitly. Then the optimal separating problem was modeled as

(12) $$\begin{array}{c} f\left(x\right)=\overset{n}{\underset{i=1}{\sum }}\alpha_{i}y_{i}K\left(x_{i},x\right)+b. \end{array}$$

And the classifier takes the form

(13) $$\begin{array}{c} y\left(x\right)=\mathrm{sign}\left[f\left(x\right)\right]. \end{array}$$

After training the model, a test sample *x* ∈ *R* ^*m*^ will be assigned to a risk type according to the following decision function:

(14) $$\begin{array}{c} y\left(x\right)=\left\{\begin{array}{cc} 1, & \text{if}\;\;f\left(x\right)>0,\\ 2, & \text{if}\;\;f\left(x\right)\leq 0. \end{array}\right. \end{array}$$

When *y*(*x*) is 1, it means that the test sample *x* is the high-risk type of HPV; otherwise, *x* should be low-risk type. Here, we selected the parameters for the sake of getting the highest overall prediction as possible. A simple grid search strategy based on 10-fold cross-validation for each dataset was performed to get the optimal values of *α*_*i*_ and *b* for prediction algorithm.

## 3. Results and Discussion

### 3.1. Evaluation Measures

Subsampling test, independent dataset test, and jackknife test are three widely used cross-validation methods to evaluate prediction's capability. The jackknife test always yields a unique outcome, which facilitates examining the quality of various predictors. Hence, we chose jackknife test to evaluate the performance of the proposed method and introduced the accuracy for each class, overall accuracy, and *F*1-score as standard performance measures, which were defined as follows:

(15) specificityaccuracy  of  high−risk  type=aa+c,sensitivityaccuracy  of  low−risk  type=db+d,accuracy  of  totality=a+da+b+c+d·100%,F1-score=2·a/a+b·a/a+ca/a+b+a/a+c·100%,=2a22a2+ac+ab·100%,

where *a* is the number of true positives, *c* is the number of false positives, *d* is the number of true negatives, and *b* is the number of false negatives. From their definition, it is interesting to note that *F*1-score will be higher if *a* is bigger. That is to say *F*1-score will be better to reflect the efficiency of HPV risk type prediction capacity.

### 3.2. Comparison of Early and Late Proteins' Performances in HPV Type Prediction

**Figure 1:**
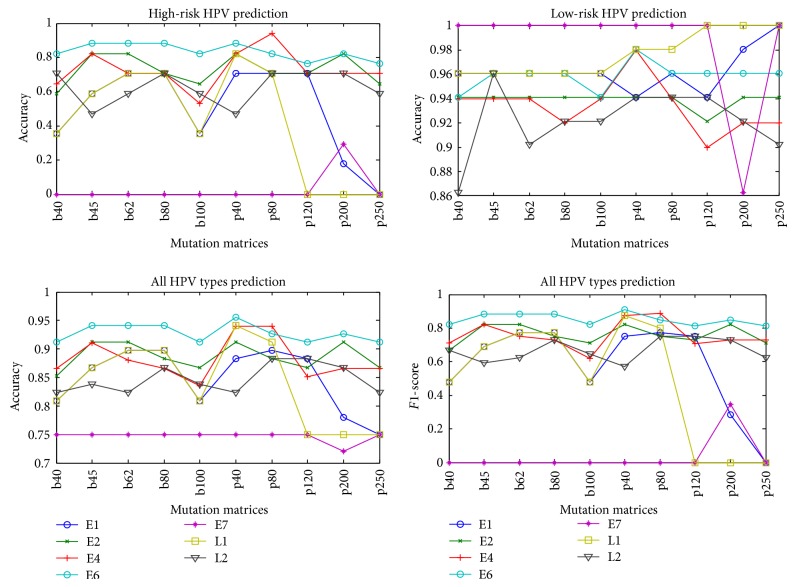
Comparison of prediction accuracy of each class, overall accuracy, and *F*1-score of all the early and late proteins. The mutation matrices in *X*-coordinate are BLOSUM 40, BLOSUM 45, BLOSUM 62, BLOSUM 80, BLOSUM 100, PAM 40, PAM 80, PAM 120, PAM 200, and PAM 250.

The HPV genome encodes a number of early (E1, E2, E4, E5, and E6) and late (L1 and L2) proteins [3, 5]. Several methods classified the high-risk and low-risk HPVs using the information from protein sequences, secondary structure, and pseudo amino acid composition [23–28]. But most of them used E6, E7, or L1 proteins. In this study, we constructed seven protein datasets of E1, E2, E4, E6, E7, L1, and L2 and compared their performance in HPV type prediction. The proteins of E5 were not included because their lengths are too small. The accuracy of each class, overall accuracy, and *F*1-score of all the early and late proteins were summarized in Figure 1.

From Figure 1, it is easy to observe that the accuracies of low-risk type are higher than that of high-risk type. For the low-risk type prediction experiment, E7 performs better than other HPV proteins expect for mutation matrix p200. But as for high-risk type prediction and all-type prediction experiments E6 achieves the best performance among all the HPV proteins according to the accuracies and *F*1-scores. Some experiment studies have shown that E5, E6, and E7 proteins of high-risk HPV play an important role in disease progression and cancer [14]. E5 protein enhances half-life and activity of epidermal growth factor receptor (EGFR). E6 and E7 proteins inactivate p53 and Rb functions [42]. The results also highlight that the sequences of E6 protein are more suitable for HPV high-risk type prediction and E7 protein is more reliable for HPV low-risk type prediction in the proposed model.

### 3.3. Comparison of Mutation Matrices in HPV Type Prediction

The proposed word statistical model was constructed based on protein “sequence space” that relies heavily on the mutation matrix. In order to evaluate the influence of different mutation matrices, we adopted ten mutation matrices including PAM 40, PAM 80, PAM 120, PAM 200, PAM 250, BLOSUM 40, BLOSUM 45, BLOSUM 62, BLOSUM 80, and BLOSUM 100. The accuracy of each class, overall accuracy, and *F*1-score of the proposed prediction method based on ten mutation matrices were represented in Figure 1.

Figure 1 largely confirms that the proposed prediction method possesses different performances based on the different mutation matrices. The changes of high-risk type and all-type prediction experiments are similar, but there is a bit of difference in the low-risk type prediction experiments. As for the BLOSUM mutation matrices, BLOSUM 45 and BLOSUM 62 perform better in the prediction of high-risk type of HPVs. For PAM mutation matrices, PAM 40 and PAM 80 achieve the better performance in the high-risk type prediction experiments. Judging from prediction accuracy, it is easier to recognize that PAM 40 achieves the best performance based on E6 protein among PAM and BLOSUM matrices except for PAM 80 with E4 protein. These results maybe give us some suggestion on how to choose a suitable mutation matrix for the prediction of high-risk type of HPVs based on the different protein sequences.

### 3.4. HPV Classification

In this study, we extracted the information using the word statistical model of E6 protein “sequence space” that was constructed based on PAM 40 mutation matrix. Leave-one-out cross-validation was applied to determine the prediction performance for all experimental results. HPV types were grouped into two classes, high-risk and low-risk.  Table 2 shows the comparison of the manually tagged answer and the results from the proposed prediction approach.

Table 2 shows that the proposed prediction method achieves better performance, in which the prediction results of 65 HPV types are consistent with their real risk types. HPV 66 and HPV 72 are high-risk types, but they are predicted as low-risk type, and HPV 30 is low-risk type, but predicted as high-risk type using the proposed prediction method. In order to highlight the prediction differences, we further compared our results with Kim's results [13]. As for Kim's prediction, HPV 72 was predicted as possible high-risk type, but it was predicted as “low-risk type” in the proposed method; HPV 56 was predicted as possible high-risk type, while we predicted it as high-risk type; HPV 53 and HPV 73 were predicted as possible high-risk types, but they are low-risk types in our results. Phylogenetic analysis showed that HPV 30 was grouped closely with the established carcinogenic type HPV 56, which indicates that HPV 30 is more likely high-risk type. From the comparison, it is easy to note that the results obtained with the proposed method are more consistent with the real risk types.

**Figure 2:**
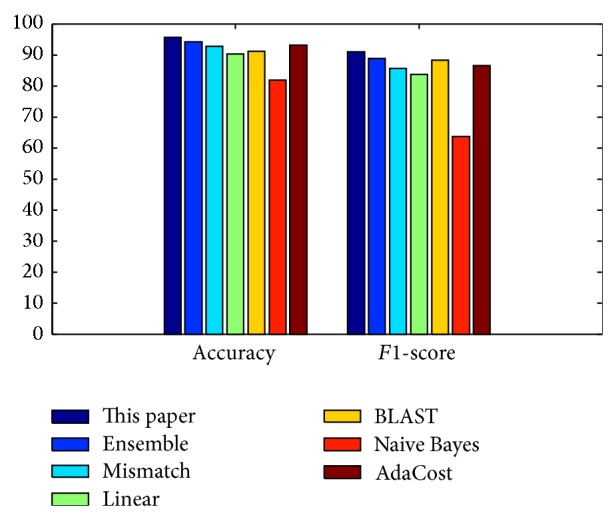
Comparison of overall accuracy and *F*1-score of all the evaluated prediction methods for HPV high-risk viral types.

To further evaluate the performance of the proposed prediction method, we computed the overall accuracy and *F*1-score and compared them with the published results in Figure 2. The methods evaluated here are as follows: SVM using Mismatch kernels (Mismatch) that have been reported in Joung et al. [24], SVM with Linear kernel method (Linear) [13], SVM classifier with the Gap-spectrum kernel (Gap) [7], BLAST predictions with a slight modification of the *k*-nearest neighbor method [13], Ensemble SVM (Ensemble) based on protein secondary structures [13], and two text-based prediction methods AdaCost [26] and nave Bayes [26].

The proposed approach achieved 95.59% accuracy and 90.91% *F*1-score, while the Ensemble SVMs obtained 94.12% accuracy and 88.89% *F*1-score, and SVM with Mismatch kernel achieved 92.70% accuracy and 85.70% *F*1-score, and SVM using Linear kernel with 90.28% accuracy and 83.72% *F*1-score and BLAST with 91.18% accuracy and 88.24% *F*1-score. As for text-based prediction method, AdaCost [26] achieved better performance with 93.05% accuracy and 84.490 % *F*1-score, and Naive Bayes [26] lags behind with 81.94% accuracy and 63.64% *F*1-score. According to the prediction accuracy and *F*1-score, the proposed prediction method achieved the best performance among all the evaluated prediction methods; the next best prediction approach is Ensemble SVMs, and the others lag behind. It is worth mentioning that the proposed approach is based on protein sequences as well as Mismatch, Linear, and Gap, while Ensemble uses the information from the predicted protein secondary structures. We also noted that text-based prediction does not provide superior results compared with other prediction methods. Although text-based prediction methods have an advantage in having explicit key words in the document, but they rely on the evidence obtained from the literature. When there is no available document for HPVs with unknown risk type, it is impossible to predict them. This comparison also indicates that the proposed word statistical model based on protein “sequence space” is more effective to classify risk types of human papillomaviruses.

### 3.5. Prediction for Unknown HPV Types

The most important task of this paper is to predict high-risk types of new HPV. Here, we downloaded the E6 protein sequences of HPVs with unknown types from the LANL database and used them to further evaluate the performance of the proposed approach.  Table 3 shows the prediction results of all the HPVs with unknown types.

From Table 3, HPV 26 and HPV 70 were predicted as high-risk types and HPV 54 and HPV 57 as low-risk types using the proposed method. In order to compare with the existing methods, we also represented the prediction results of available approaches in Table 3. From the HPV classification, we knew that the proposed prediction method achieved the best performance, and it is followed by Ensemble SVMs. From Table 3, it is easy to note that the proposed method and Ensemble SVMs achieve the same results. As for the HPV 54 and HPV 57, all the methods predicted them as low-risk types. For HPV 26, the proposed method, PseAAC [27], Ensemble [13], and Gap [7] predicted it as high-risk type, while Mismatch [24], Linear [13], and Genetic [23] predicted it as low-risk type. According to the reliabilities of the prediction approaches, HPV 26 should be high-risk type. As for HPV 70, all the prediction methods predicted it as high-risk type except for Genetic [23] and PseAAC [27]. These results show that the proposed method can provide a simple but efficient guideline for the investigation of potentially high-risk HPVs.

## 4. Conclusion

Genital human papillomaviruses have a strong relationship with cervical cancer, especially high-risk viral types of HPVs. Therefore, discrimination of HPV risk type plays an important role in the diagnosis and remedy of cervical cancer. This paper proposed a computational scheme to predict high-risk types of HPVs with word statistical model of protein “sequence space.” With help of mutation matrices, we first constructed a sequence space of the given protein sequences. Instead of only using sequence-based or structure-based information of protein sequences, we extracted the information of HPV from the protein “sequence space” with word statistical model to predict high-risk types of HPVs. The proposed method was tested on 68 samples with known HPV types and 4 samples with unknown HPV types. The results show that the proposed method achieved better performance in comparison to the previous methods.

The main goal of our research is to investigate a new prediction method based on protein “sequence space.” The first contribution can be seen from comparison of early and late proteins' performances in HPV type prediction; we found that the “sequence space” of E6 protein is more suitable for HPV high-risk type prediction, while that of E7 protein is more reliable protein for HPV low-risk type prediction. The second contribution can be indicated from comparison of mutation matrices in HPV type prediction; we noticed that PAM 40 achieves the best performance with the sequences of E6 protein among PAM and BLOSUM matrices except for PAM 80 with E4 protein. The third contribution can be deduced from HPV classification and prediction for unknown HPV types; we found that the proposed prediction method achieved the best performance among all the evaluated prediction methods, with 95.59% accuracy and 90.91% *F*1-score, which can be contributed to the introduction of the protein “sequence space.” Thus, this understanding can be used to guide development of more powerful method for prediction of high-risk types of HPVs.

## Figures and Tables

**Table 1:** Star sets of 20 amino acids based on PAM250 substitution matrix.

Matrix	Star set				

PAM250	{AGPST}	{C}	{DEGHNQ}	EDHNQ	{FILY}
	{GADS}	{HDENQR}	{IFLMV}	{KNQR}	{LFIMV}
	{MILV}	{NDEHKQS}	{PAS}	{QDEHKNR}	{RHKQW}
	{SAGNPT}	{TAS}	{VILM}	{WR}	{YF}

**Table 2:** Comparison of the real risk types (REAL) and the prediction results using the proposed approach.

Types	Real	Predicted	Types	Real	Predicted	Types	Real	Predicted	Types	Real	Predicted
HPV 39	High	High	HPV 7	Low	Low	HPV 34	Low	Low	HPV 50	Low	Low
**HPV 72**	**High**	**Low**	**HPV 30**	**Low**	**High**	HPV 44	Low	Low	HPV 5	Low	Low
HPV 33	High	High	HPV 73	Low	Low	HPV 43	Low	Low	HPV 20	Low	Low
HPV 51	High	High	HPV 6	Low	Low	HPV 32	Low	Low	HPV 23	Low	Low
HPV 16	High	High	HPV 27	Low	Low	HPV 24	Low	Low	HPV 19	Low	Low
HPV 56	High	High	HPV 13	Low	Low	HPV 8	Low	Low	HPV 47	Low	Low
HPV 18	High	High	HPV 55	Low	Low	HPV 48	Low	Low	HPV 22	Low	Low
HPV 59	High	High	HPV 2	Low	Low	HPV 12	Low	Low	HPV 25	Low	Low
HPV 52	High	High	HPV 10	Low	Low	HPV 49	Low	Low	HPV 9	Low	Low
HPV 35	High	High	HPV 42	Low	Low	HPV 15	Low	Low	HPV 36	Low	Low
HPV 68	High	High	HPV 28	Low	Low	HPV 21	Low	Low	HPV 41	Low	Low
HPV 58	High	High	HPV 40	Low	Low	HPV 4	Low	Low	HPV 63	Low	Low
HPV 31	High	High	HPV 3	Low	Low	HPV 65	Low	Low	HPV 1	Low	Low
**HPV 66**	**High**	**Low**	HPV 11	Low	Low	HPV 37	Low	Low	HPV 80	Low	Low
HPV 45	High	High	HPV 29	Low	Low	HPV 38	Low	Low	HPV 77	Low	Low
HPV 61	High	High	HPV 74	Low	Low	HPV 60	Low	Low	HPV 76	Low	Low
HPV 67	High	High	HPV 53	Low	Low	HPV 17	Low	Low	HPV 75	Low	Low

**Table 3:** Prediction results of the HPVs with unknown types using the proposed prediction methods and from the available methods.

Types	Prediction methods						
	Mismatch [24]	Linear [13]	Gap [7]	Genetic [23]	PseAAC [27]	Ensemble [13]	This paper
HPV 26	Low	Low	High	Low	High	High	High
HPV 54	Low	Low	Low	Low	Low	Low	Low
HPV 57	Low	Low	Low	Low	Low	Low	Low
HPV 70	High	High	High	Low	Low	High	High
